# Supplementary material for: Serum Uric Acid Levels in Parkinson’s Disease: A Cross-Sectional Electronic Medical Record Database Study from a Tertiary Referral Centre in Romania
Source: Medicina (Kaunas). 2022 Feb 6;58(2):245. doi: 10.3390/medicina58020245 (PMC8877142; doi:10.3390/medicina58020245)
Supplement: Supplementary file 1 [file medicina-58-00245-s001.zip › medicina-1547123-supplementary.pdf]

Supplementary Table S1. Spreadsheet of the Parkinson`s disease patients included in the study - demographic data, presence of motor complications, symptomatic treatment

Patient no.	Sex	Age (years)	PD duration	Age at onset	Motor complications	Levodopa	DA	IMAO	COMT inhibitors	LEDD (mg)	Psychiatric medication
**1**	M	60	9	51	no	yes	yes	yes	no	204	no
**2**	M	65	4	61	no	no	yes	yes	no	160	no
**3**	F	71	3	68	no	yes	no	yes	no	475	no
**4**	M	67	15	52	yes	yes	no	yes	no	2095	yes
**5**	M	66	14	52	no	yes	no	yes	yes	1023	yes
**6**	F	70	3	67	no	yes	yes	yes	no	529	no
**7**	M	71	5	66	no	no	yes	yes	no	310	no
**8**	F	67	2	65	no	yes	no	yes	no	850	no
**9**	M	56	4	52	no	no	yes	yes	no	120	no
**10**	M	60	1	59	no	yes	yes	no	no	1157	no
**11**	M	60	0	60	no	yes	yes	yes	no	515	no
**12**	F	74	4	70	no	yes	no	yes	no	350	yes
**13**	M	60	4	56	no	yes	yes	yes	no	1079	yes
**14**	M	78	NA	NA	no	yes	no	no	no	475	yes
**15**	M	56	NA	NA	no	yes	yes	yes	no	1170	yes
**16**	M	72	1	71	no	yes	no	no	no	375	no
**17**	M	63	13	50	yes	yes*	no	no	no	839	yes
**18**	M	65	1	64	no	no	yes	yes	no	120	no
**19**	M	69	11	58	yes	yes	yes	no	yes	1378	yes
**20**	F	87	NA	NA	no	yes	no	no	no	375	no
**21**	M	69	2	67	no	no	yes	yes	no	180	no
**22**	F	67	NA	NA	yes	yes*	no	no	no	2593	yes
**23**	F	71	NA	NA	yes	yes	no	yes	yes	726	yes
**24**	F	63	NA	NA	yes	yes	yes	yes	yes	2008	yes
**25**	F	62	NA	NA	yes	yes	yes	yes	yes	1679	yes
**26**	M	66	NA	NA	no	yes	no	no	no	250	no
**27**	M	74	4	70	yes	yes	yes	yes	yes	1014	no
**28**	M	55	0	55	no	yes	no	yes	no	725	yes
**29**	M	85	5	80	no	yes	yes	yes	no	1610	yes
**30**	M	74	NA	NA	no	yes	no	yes	no	1100	no
**31**	M	54	4	50	yes	yes	yes	yes	no	1545	no
**32**	M	83	NA	NA	no	yes	no	yes	no	1100	yes
**33**	F	70	6	64	yes	yes*	no	no	no	1242	yes
**34**	M	79	NA	NA	no	yes	yes	no	yes	1570	yes
**35**	M	67	NA	NA	no	yes	yes	yes	no	670	yes
**36**	F	76	NA	NA	yes	yes	no	yes	yes	1348	yes
**37**	M	81	2	79	no	yes	no	no	no	250	yes
**38**	F	70	10	60	yes	yes	yes	yes	yes	1398	yes
**39**	M	73	23	50	yes	yes*	no	yes	no	968	yes
**40**	F	80	NA	NA	no	yes	no	yes	no	600	yes
**41**	F	74	7	67	yes	yes	yes	no	yes	1864	no
**42**	F	80	1	79	no	yes	no	yes	no	850	no
**43**	M	75	2	73	no	yes	no	no	no	500	no
**44**	M	79	4	75	NA	NA	NA	NA	NA	NA	NA
**45**	M	78	NA	NA	NA	NA	NA	NA	NA	NA	NA
**46**	M	51	1	50	no	NA	NA	NA	NA	NA	NA
**47**	M	67	NA	NA	NA	NA	NA	NA	NA	NA	NA
**48**	M	54	4	50	yes	yes	yes	yes	no	1545	no
**49**	M	70	1	69	NA	NA	NA	NA	NA	NA	NA

(COMT = catechol O-methyl transferase; DA = dopamine agonists; F = female; LEDD = levodopa equivalent daily dose; M = male; MAOI = monoaminoxidase inhibitors; NA = not available; * = levodopa/carbidopa intestinal gel, included in the LEDD)

Supplementary Table S2. Spreadsheet of the Parkinson`s disease patients included in the study - Hoehn and Yahr stage, presence of cerebrovascular disease, cognitive impairment, and serum level of uric acid

Patient no.	Hoehn and Yahr stage	Cerebrovascular disease	Cognitive impairment	Serum uric acid (3.4-7 mg/dl)
**1**	NA	yes	yes	6.7
**2**	3	NA	NA	5.2
**3**	2	yes	no	6.4
**4**	3	yes	yes	3.3
**5**	3	no	yes	4.6
**6**	3	no	no	3.9
**7**	1	no	no	5.7
**8**	2	NA	NA	5.8
**9**	2	no	no	5.9
**10**	2	no	no	6.8
**11**	2	NA	NA	5.9
**12**	2	NA	NA	3.1
**13**	2.5	NA	NA	4.9
**14**	2	yes	yes	5.1
**15**	2	no	no	5.9
**16**	NA	NA	NA	5.9
**17**	4	no	yes	4.1
**18**	2	no	no	5.9
**19**	2	yes	yes	5.6
**20**	NA	yes	no	7.5
**21**	2	no	no	5.8
**22**	4	no	yes	4
**23**	3	yes	yes	3.9
**24**	3	no	no	3.3
**25**	4	no	no	4.1
**26**	NA	no	no	4.3
**27**	3	no	yes	3.7
**28**	NA	no	no	7.3
**29**	2.5	NA	no	6.5
**30**	NA	no	no	5.7
**31**	2	no	no	6.9
**32**	3	yes	yes	4.9
**33**	NA	NA	yes	3.2
**34**	NA	no	yes	5.4
**35**	3	no	no	4.2
**36**	3	no	no	3.2
**37**	2	yes	yes	5.1
**38**	3	no	no	7.5
**39**	4	no	yes	3.6
**40**	3	yes	yes	2.7
**41**	4	yes	no	3.5
**42**	2	yes	no	3.5
**43**	2	yes	no	5.5
**44**	3	no	yes	4
**45**	NA	NA	NA	5.3
**46**	1	NA	NA	3.9
**47**	2	no	no	4.3
**48**	2	no	no	5.3
**49**	NA	no	NA	5.9

(NA = not available)

Supplementary Table S3. Results of logistic regression analysing relationship between serum uric acid and the presence or absence of motor complications and cognitive impairment

Dependent variable	Unstandardized regression weight	P value	OR	95% CI for OR	
				Lower	Upper
Motor complications	- 0.595	0.034	0.552	0.318	0.957
Cognitive impairment	- 0.617	0.028	0.539	0.310	0.957

(OR = odds ratio; CI = confidence interval)

Supplementary Table S4. Results of the Kolmogorov-Smirnov and Shapiro-Wilk tests performed to evaluate distribution of age and serum uric acid level, in SPSS® Statistics Subscription.

Tests of Normality

	Kolmogorov-Smirnov[a]			Shapiro-Wilk		
	Statistic	df	Sig.	Statistic	df	Sig.
Age	.059	49	.200*	.985	49	.777
Serum UA	.115	49	.118	.962	49	.111

*. This is a lower bound of the true significance.

a. Lilliefors Significance Correction; UA = uric acid

(UA = uric acid)

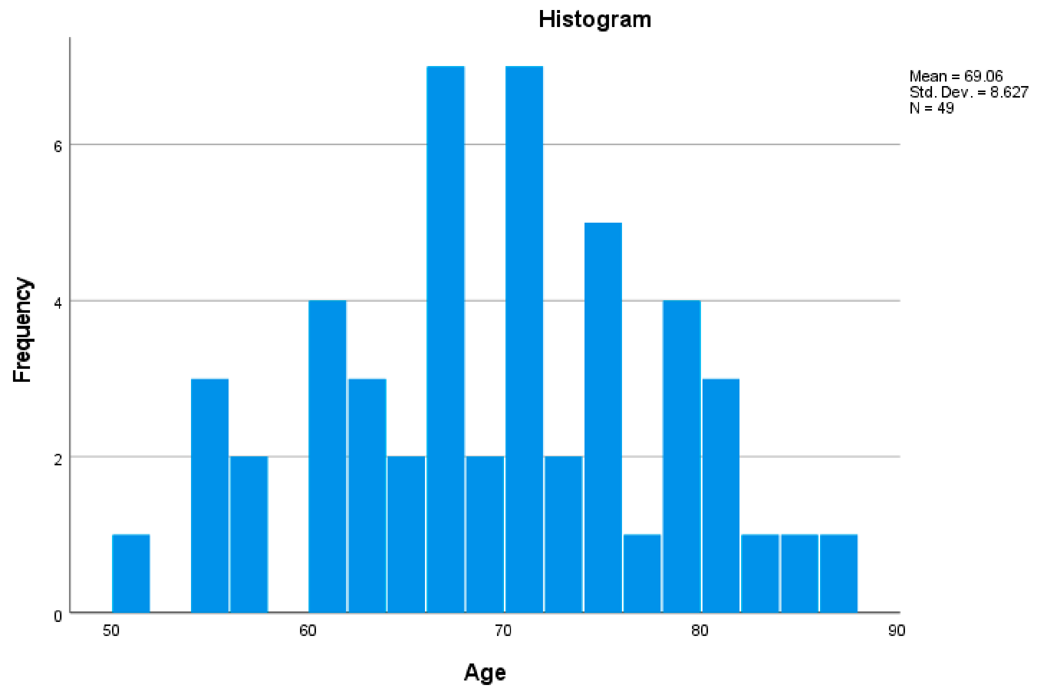

Supplementary Figure S1. Histogram showing normality of age for the included Parkinson`s disease patients, in SPSS® Subscription

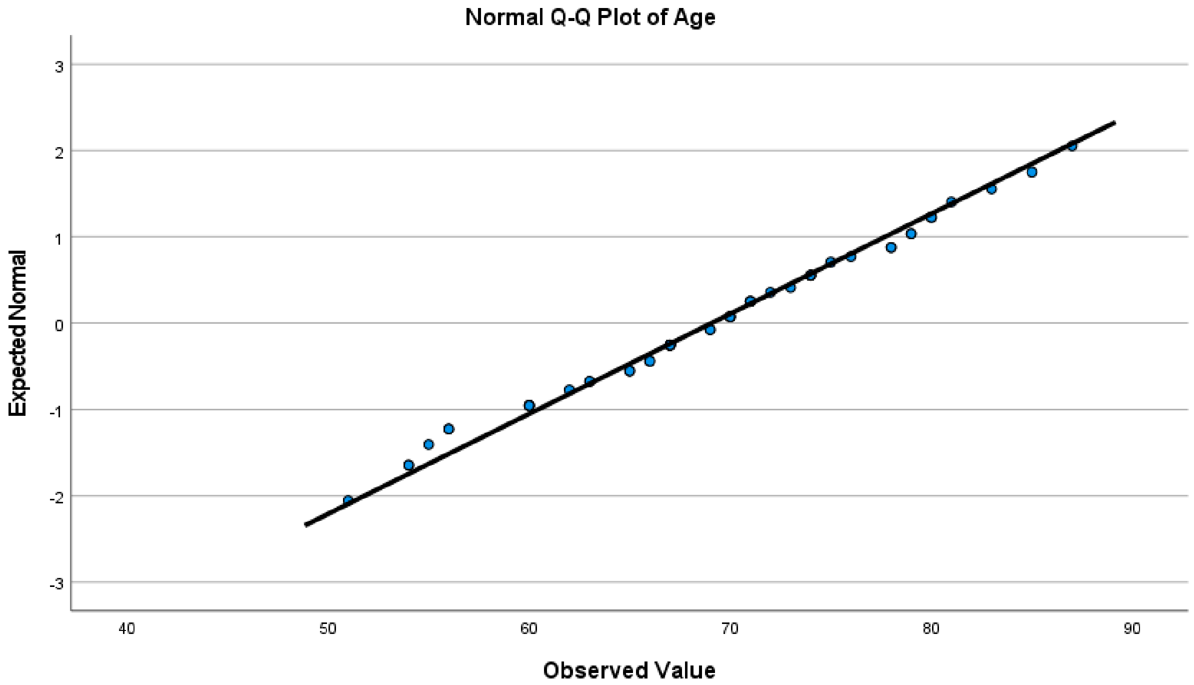

Supplementary Figure S2. Normal Q-Q plot showing normality of age for the included Parkinson`s disease patients, in SPSS® Subscription

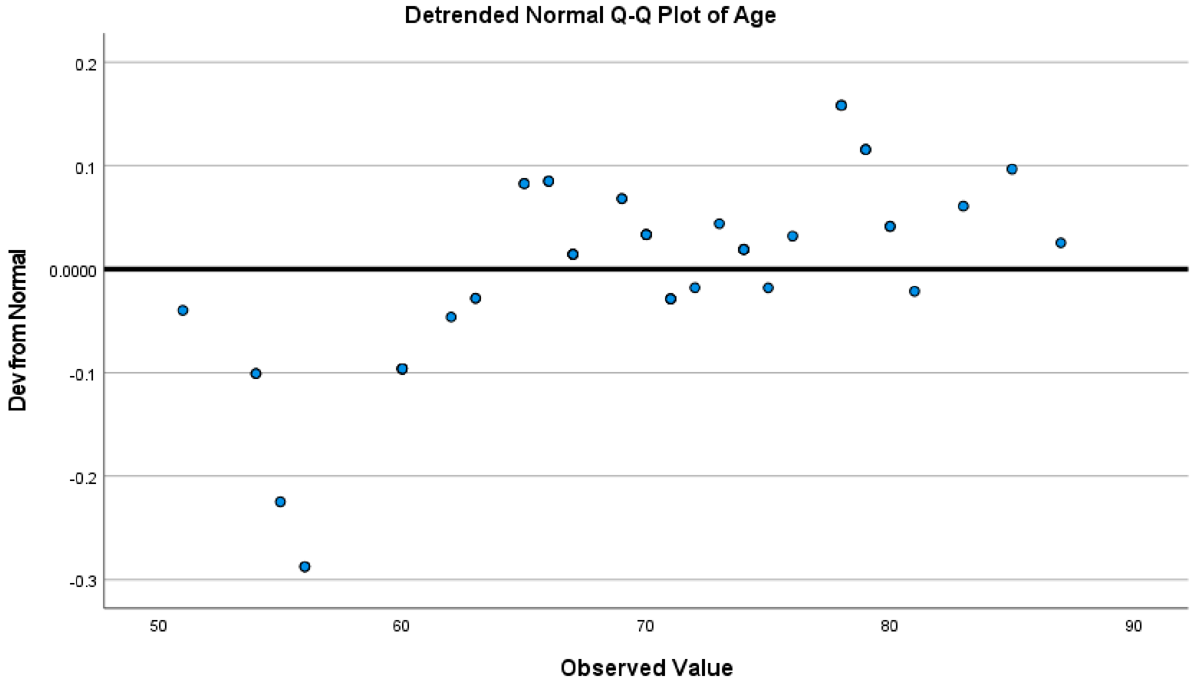

Supplementary Figure S3. Detrended normal Q-Q plot showing normality of age for the included Parkinson`s disease patients, in SPSS® Subscription

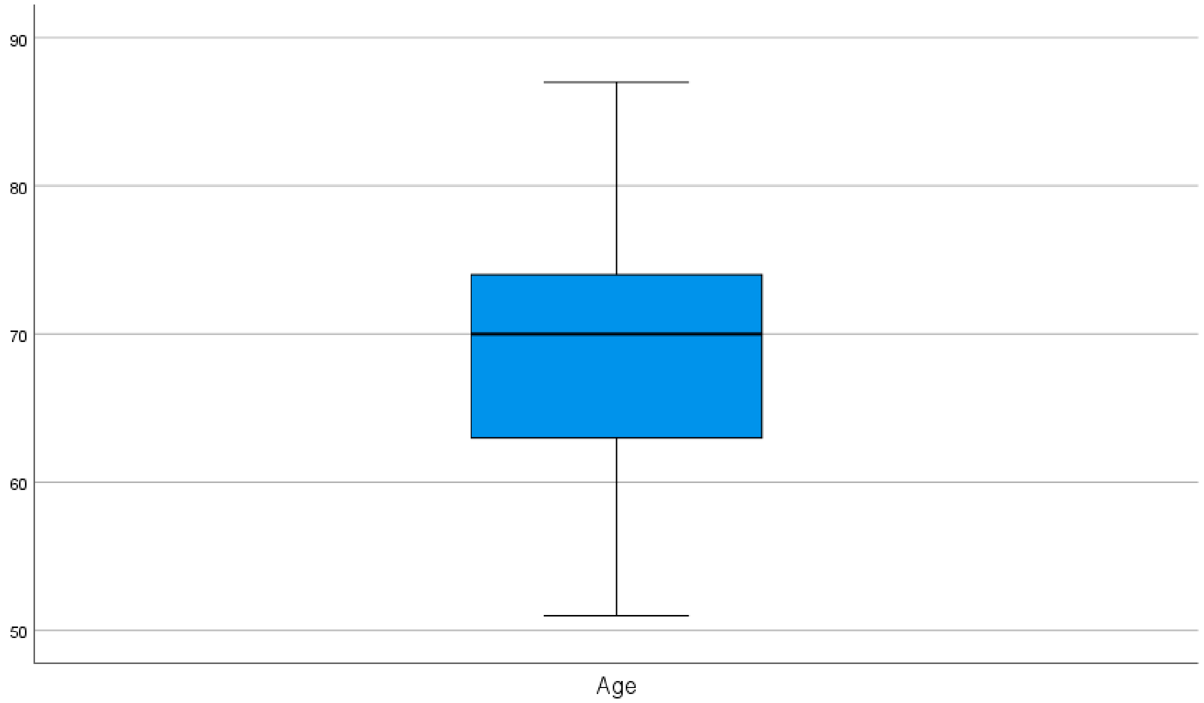

Supplementary Figure S4. Boxplot showing normality of age for the included Parkinson`s disease patients, in SPSS® Subscription

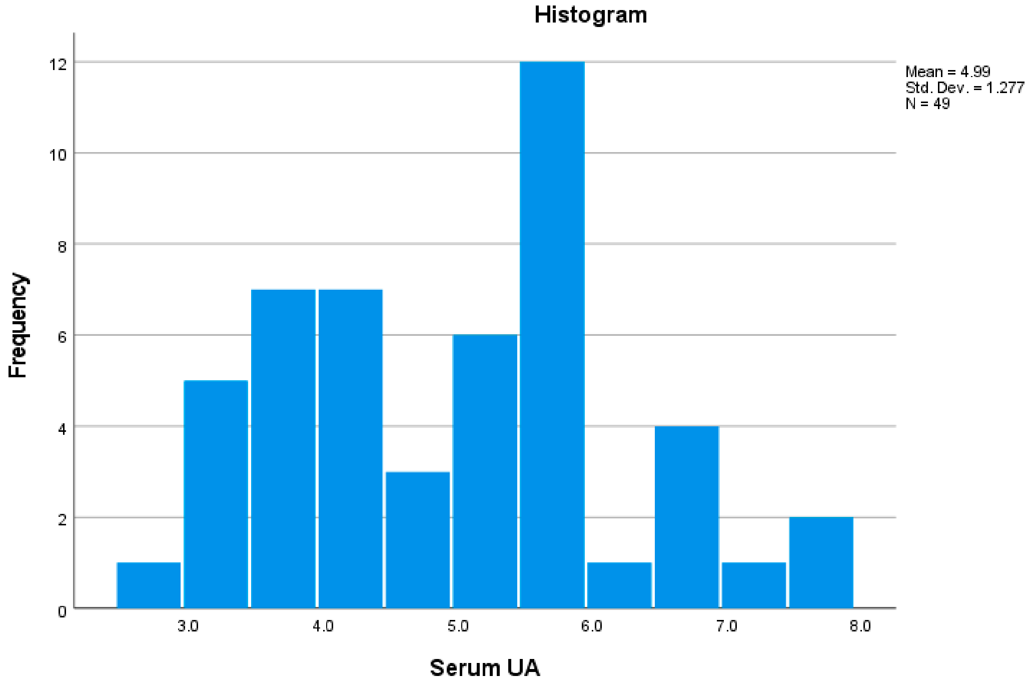

Supplementary Figure S5. Histogram showing normality of serum uric acid for the included Parkinson`s disease patients, in SPSS® Subscription
(UA = uric acid)

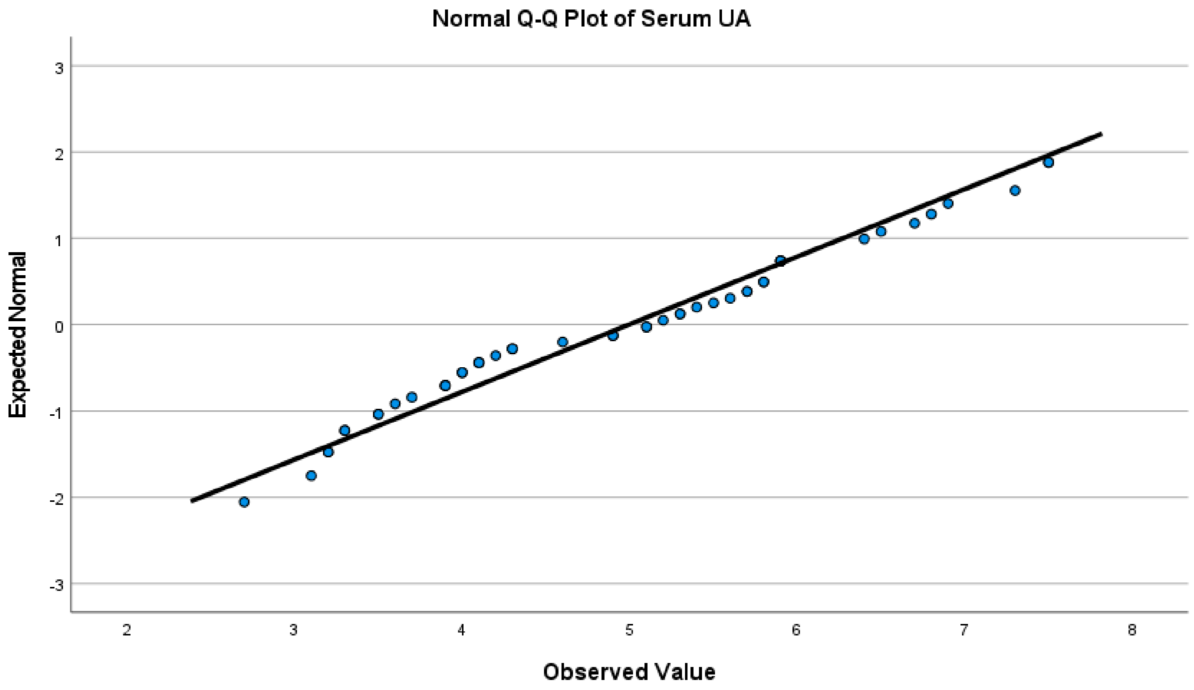

Supplementary Figure S6. Normal Q-Q plot showing normality of serum uric acid for the included Parkinson`s disease patients, in SPSS® Subscription
(UA = uric acid)

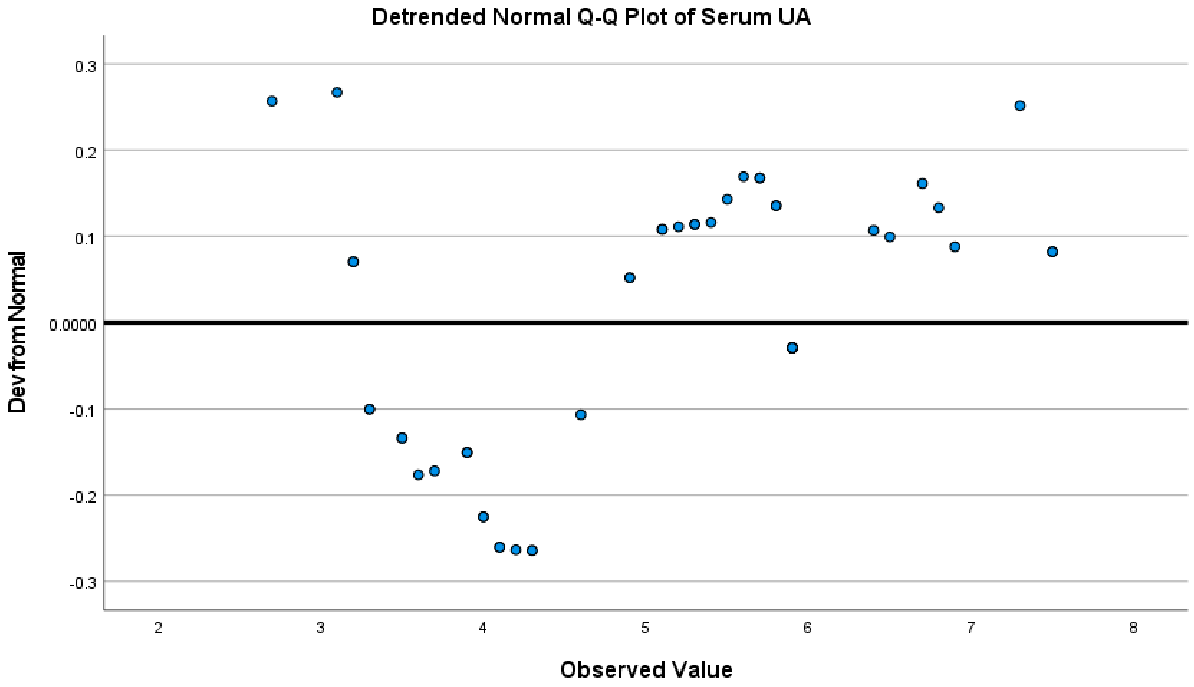

Supplementary Figure S7. Detrended normal Q-Q plot showing normality of serum uric acid for the included Parkinson`s disease patients, in SPSS® Subscription
(UA = uric acid)

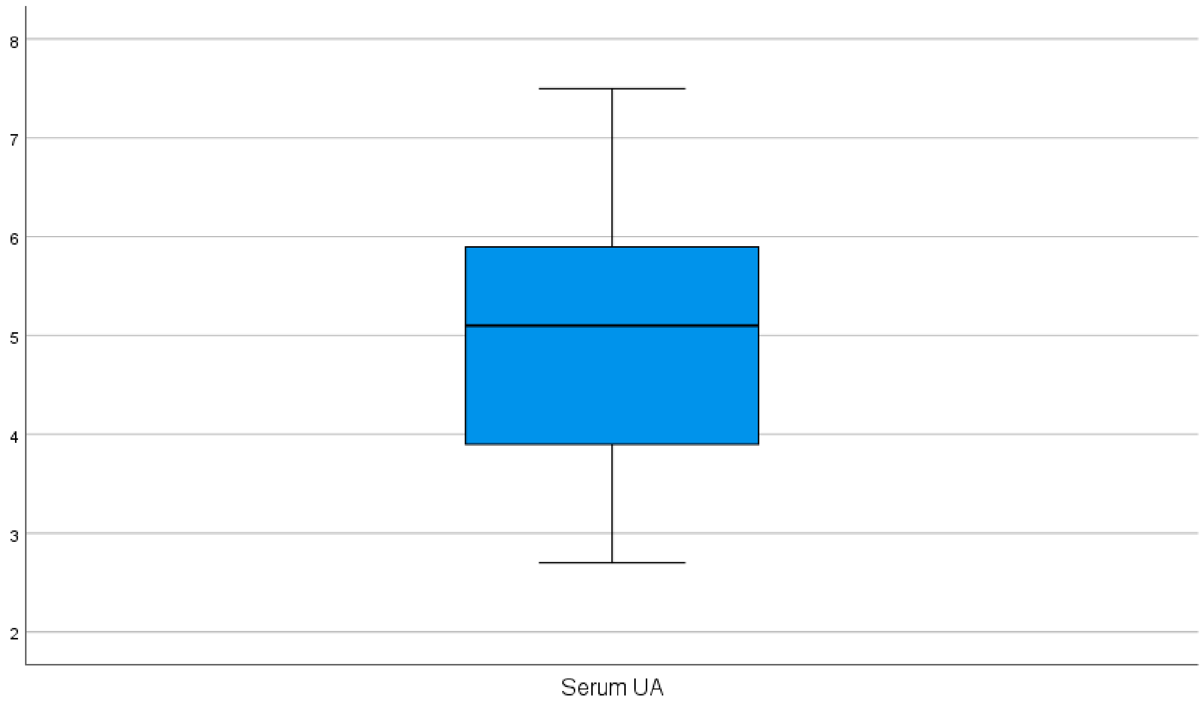

Supplementary Figure S8. Boxplot showing normality of age for the included Parkinson`s disease patients, in SPSS® Subscription
(UA = uric acid)

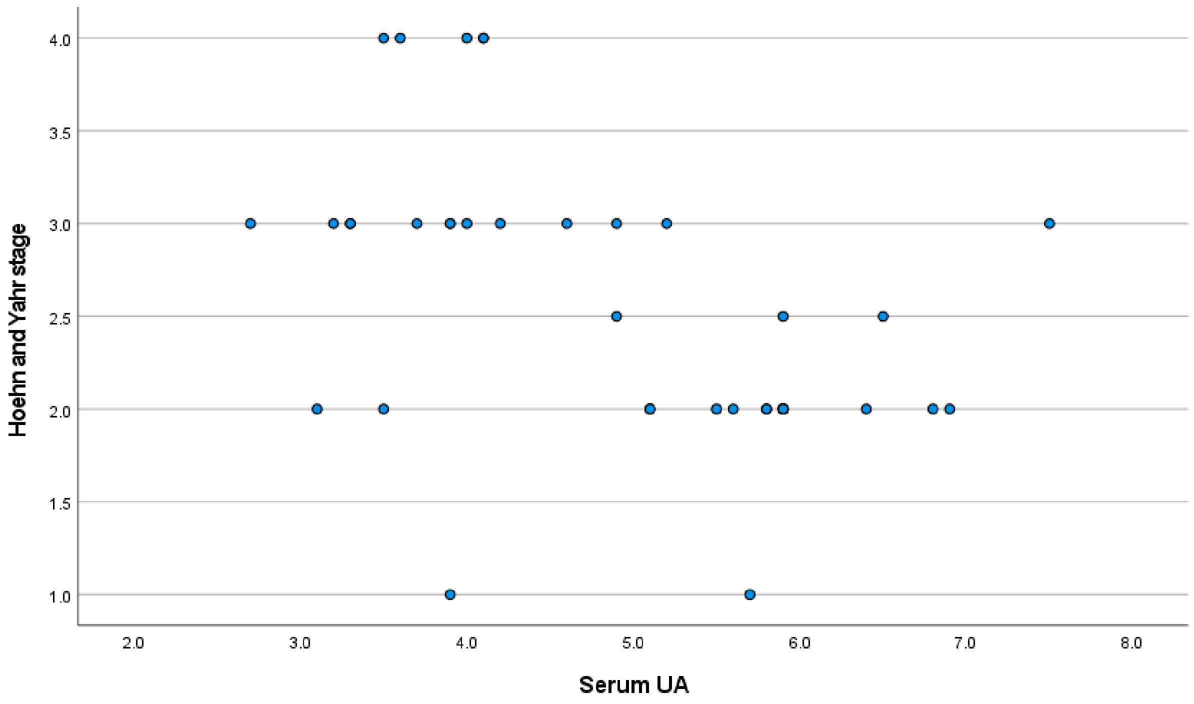

Supplementary Figure S9. Spearman graph showing correlation between lower level of serum uric acid and the Hoehn and Yahr stage.
(UA = uric acid)

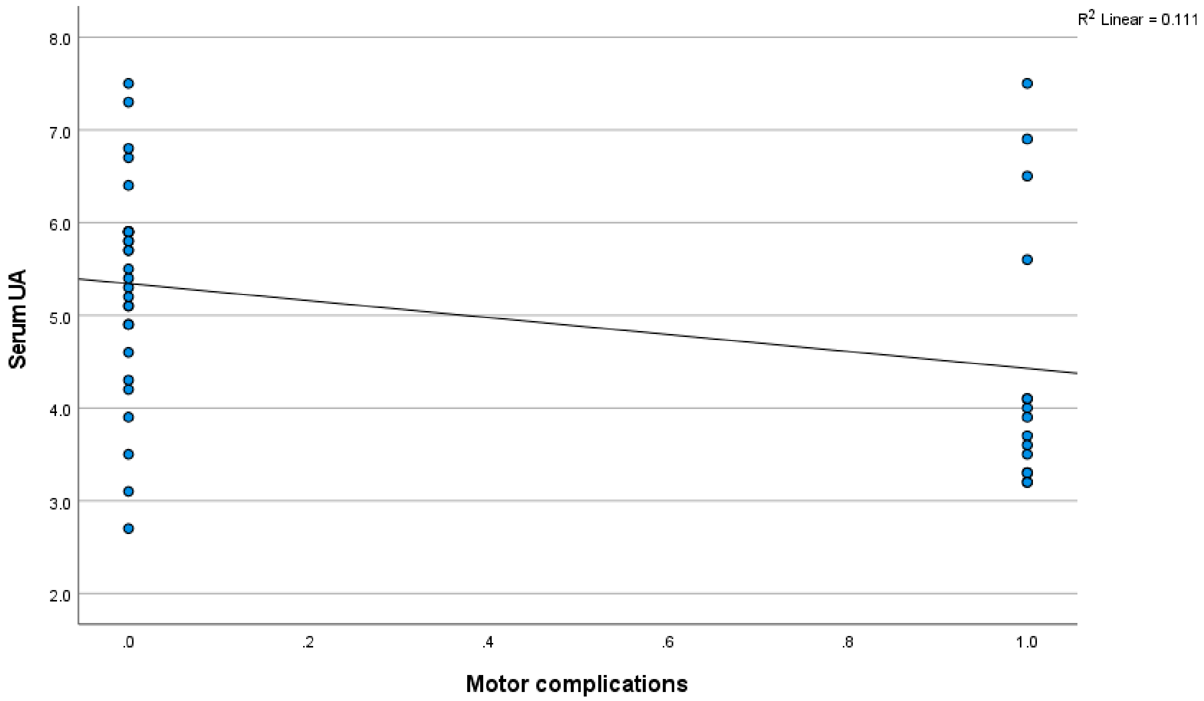

Supplementary Figure S10. Point-biserial correlation graph showing association between lower level of serum uric acid and presence of motor complications.
(UA = uric acid)

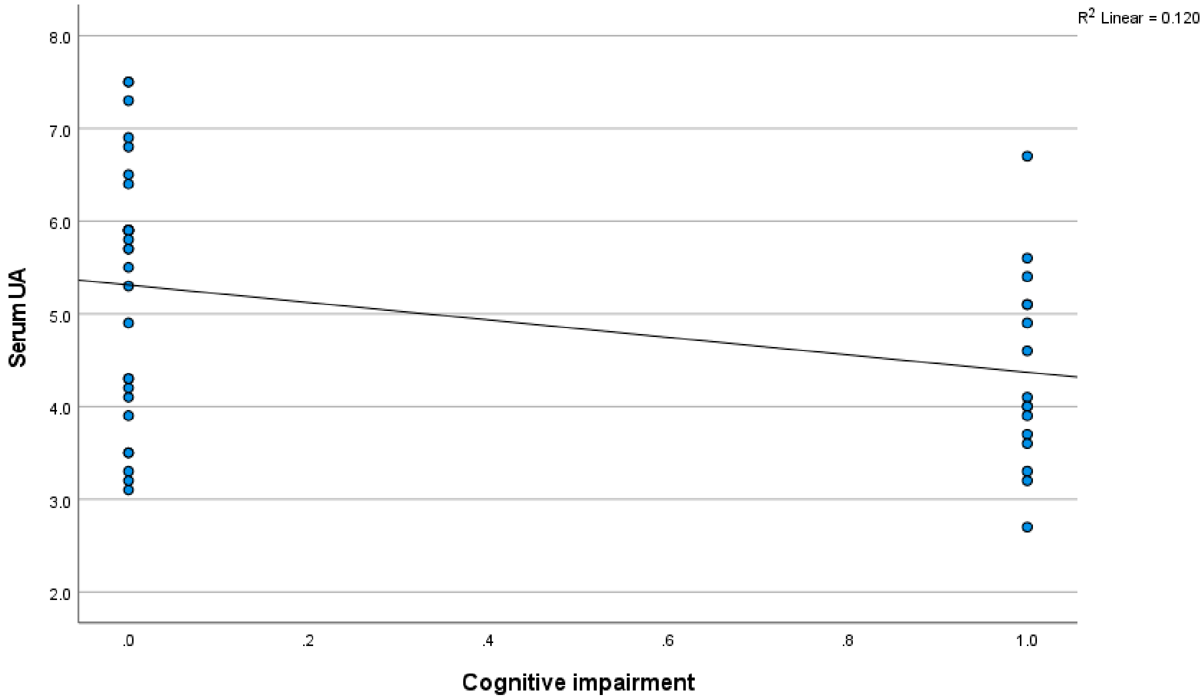

Supplementary Figure S11. Point-biserial correlation graph showing association between lower level of serum uric acid and presence of cognitive impairment.
(UA = uric acid)
